# Does fish oil supplementation increase cholesterol efflux capacity in familial hypercholesterolaemia?

**DOI:** 10.1111/eci.14048

**Published:** 2023-06-29

**Authors:** Dick C. Chan, Annalisa Ronca, Qidi Ying, Jing Pang, Mikaël Croyal, Gerald F. Watts, Elda Favari

**Affiliations:** ^1^ Medical School, Faculty of Health and Medical Sciences University of Western Australia Perth Western Australia Australia; ^2^ Department of Food and Drug University of Parma Parma Italy; ^3^ Nantes Université CNRS, INSERM, l'institut du thorax Nantes France; ^4^ Nantes Université CHU Nantes, Inserm, CNRS, SFR Santé, Inserm UMS 016 Nantes France; ^5^ CRNH‐Ouest Mass Spectrometry Core Facility Nantes France; ^6^ Lipid Disorders Clinic, Department of Cardiology and Internal Medicine Royal Perth Hospital Perth Western Australia Australia

## 1 INTRODUCTION

Familial hypercholesterolaemia (FH) is a dominantly inherited disorder principally due to mutations in the low‐density lipoprotein (LDL)‐receptor pathway that causes markedly elevated plasma LDL‐cholesterol concentration and premature coronary heart disease (CHD).^1^ In addition to elevated LDL‐cholesterol levels, there is evidence that alterations in reverse cholesterol transport (RCT) may also contribute to accelerating atherosclerosis in FH.^2^

Excess cholesterol in macrophages is eliminated via the process of RCT in which cholesterol from peripheral tissues is transported to the liver for biliary excretion.^3^ Cholesterol efflux from macrophages, the first step of RCT, plays a major role in anti‐atherogenesis. Cholesterol efflux capacity (CEC) is an ex vivo measure of plasma acceptors to accept cholesterol released from cells through different receptor‐mediated pathways, such as ATP binding cassette transporter A1 (ABCA1), ABCG1 and scavenger receptor class B type I (SR‐BI).^3^, ^4^ Recent observational studies and meta‐analysis suggest that the capacity of plasma to effect cholesterol efflux is inversely related to atherosclerotic cardiovascular disease (ASCVD), independent of traditional cardiovascular risk factors, including the levels of LDL‐cholesterol and high‐density lipoprotein (HDL)‐cholesterol.^5^

Fish oils are a rich source of long‐chain omega‐3 fatty acid (ω‐3FAs), primarily eicosapentaenoic acid (EPA) and docosahexaenoic acid (DHA).^6^ Both EPA and DHA may reduce the risk of ASCVD through various mechanisms, including triglyceride lowering, membrane stabilization and antithrombotic, anti‐inflammatory or antiarrhythmic properties. These favourable vascular effects of ω‐3FAs may contribute to improved cardiovascular outcomes, as demonstrated in large intervention trials.^7^ Whether ω‐3FA intervention promotes the RCT process in FH is unclear.

We have previously reported that a high‐dose of ω‐3FAs improved triglyceride‐rich lipoprotein metabolism in FH patients receiving standard treatment for lowering LDL‐cholesterol.^8^ In the present study, we opportunistically explored the effects of high dose ω‐3FAs on ex vivo CEC in these patients.

## 2 METHOD

Full details of patient recruitment, study design, clinical protocol and biochemical measurements and statistical analysis are given in the Supplementary Materials.^8^, ^9^, ^10^

### 2.1 Measurement of total cholesterol efflux capacity

Efflux studies were performed as previously described using J774 macrophages.^9^, ^10^ Whole plasma, apoB‐depleted plasma, ABCA1‐mediated and passive diffusion‐mediated CEC were determined through the use of specific cell condition models. Briefly, cells were seeded in DMEM with 10% of FCS and labelled with [^3^H]‐cholesterol in the presence of an ACAT inhibitor (2 μg/mL, Sandoz 58035; Sigma‐Aldrich). J774 cells were then treated with 0.3 mM cAMP analogue in 0.2% BSA for 18 h to upregulate ABCA1. The efflux medium was prepared using 2% (v/v) whole plasma (or 2.8% apoB‐depleted plasma) and incubated with cells for 4–6 h. Whole plasma (or apoB‐depleted plasma) CEC was expressed as a percentage of radiolabeled cholesterol released to the medium over the total radioactivity incorporated by cells. Using apoB‐depleted plasma, the ABCA1‐mediated CEC was calculated as the difference between the percentage efflux obtained in cAMP treated cell (i.e. apoB‐depleted plasma CEC) and that obtained in cells not treated with cAMP (i.e. passive diffusion‐mediated CEC). Measurements of ABCG1‐ and SR‐BI‐mediated CEC are given in the Supplementary Materials.

### 2.2 Statistical analyses

All data were analysed using the SPSS 21 software (SPSS). Data were presented as mean ± SEM unless otherwise indicated. The Shapiro–Wilk test was used to determine whether variables were normally distributed. Groups were compared using independent *t*‐tests. Paired *t*‐tests were used between the FH patients, after logarithmic transformation of skewed variables where appropriate. Carryover effect of the cross‐over design was estimated using SAS 9.2 (SAS Institute). Significance was defined at the 5% level using a two‐tailed test.

## 3 RESULTS

### 3.1 Baseline clinical and biochemical characteristics

Twenty patients with FH (10 men and 10 women) completed the study. On average, they were middle‐aged (53.3 ± 3.0 years), nonobese (body mass index 26.6 ± 5.8 kg/m^2^) and normotensive (systolic blood pressure 121 ± 15 mmHg and diastolic blood pressure 69 ± 8 mmHg) at screening. Seventeen patients were genetically diagnosed with FH (i.e. pathogenic *LDLR* mutations), with three having definite phenotypic FH (Dutch Lipid Clinic Network Criteria score >8). Nine subjects were on rosuvastatin, eight on atorvastatin and three on simvastatin. Of these, 13 patients were also on ezetimibe (10 mg/day). Three subjects were on anti‐hypertension medication and four reported a history of coronary artery disease (CAD) event. All patients maintained their cholesterol‐lowering or anti‐hypertension mediations during the study.

Using data from previous studies,^9^ whole plasma CEC (16.4% ± 4.0% vs. 20.0% ± 4.9%) and passive diffusion‐mediated CEC (8.1% ± 2.9% vs. 11.0% ± 1.8%) were significantly lower (*p* < 0.01 in both) in the FH patients than a group of 81 healthy normolipidemic non FH subjects (age 32 ± 11 years; body mass index 25.1 ± 2.8 kg/m^2^). ApoB‐depleted CEC was also lower in the FH patients than the non FH controls (12.9% ± 3.1% vs. 14.0% ± 2.6%), which failed to reach significance (*p* = 0.11). In contrast, ABCA1‐mediated CEC was significantly higher in the FH patients compared with the control group (4.9% ± 1.3% vs. 3.1% ± 1.6%; *p* < 0.01).

### 3.2 Treatment effect on clinical and biochemical characteristics

The effects of ω‐3FAs on the clinical and biochemical characteristics in these 20 FH patients have been previously published.^8^ As seen in Table 1, ω‐3FAs significantly lowered systolic blood pressure and diastolic blood pressure, plasma triglycerides, apoB and VLDL‐apoB concentration (*p* < 0.05 in all). Total cholesterol, LDL‐cholesterol, HDL‐cholesterol, apoA‐I, apoA‐II, apo(a) and Lp(a) concentrations were not significantly altered with ω‐3FA treatment, nor were glucose, insulin concentrations and HOMA score. Body weight, waist circumference, body mass index and pulse pressure also did not alter significantly during the intervention (*p* > 0.05 in all). There was no significant change in energy intake (7275 ± 571 kJ vs. 7068 ± 596 kJ) or energy from fat (32.1% ± 1.9% vs. 34.5% ± 2.3%), protein (18.9% ± 0.8% vs. 19.4% ± 1.1%), carbohydrate (46.5% ± 2.8% vs. 43.1% ± 2.5%), alcohol consumption (2.5% ± 0.8% vs. 3.0% ± 1.7%) and exercise expenditure (8121 ± 651 kJ vs. 7772 ± 628 kJ) between no treatment and active treatment periods with ω3‐FAs (*p* > 0.05 for all). Capsule count reported a >95% compliance, confirmed by significant elevations in circulating plasma EPA (+215%) and DHA (+64%) levels (*p* < 0.001 in both).

**TABLE 1:** Clinical and biochemical characteristics with and without ω‐3FA supplementation in 20 FH patients.

	No treatment	ω‐3FA supplementation	*p*
Weight (kg)	79.1 ± 3.6	79.0 ± 3.5	0.801
Waist circumference (cm)	90.5 ± 2.9	90.4 ± 3.1	0.884
Body mass index (kg/m^2^)	27.0 ± 1.4	27.0 ± 1.3	0.702
Systolic blood pressure (mmHg)	124 ± 3.2	117 ± 3.4	0.009
Diastolic blood pressure (mmHg)	69.3 ± 1.9	65.1 ± 1.9	0.006
Pulse pressure (mmHg)	54.9 ± 2.3	51.4 ± 2.5	0.121
Total cholesterol (mmol/L)	4.58 ± 0.27	4.20 ± 0.16	0.069
Triglycerides (mmol/L)	1.20 (0.99–1.45)	0.98 (0.83–1.17)	0.001
LDL‐cholesterol (mmol/L)	2.81 ± 0.29	2.54 ± 0.16	0.204
HDL‐cholesterol (mmol/L)	1.19 ± 0.12	1.12 ± 0.05	0.554
Non HDL cholesterol (mmol/L)	3.39 ± 0.27	3.07 ± 0.18	0.098
Apolipoprotein A‐I (g/L)	1.34 ± 0.06	1.31 ± 0.07	0.561
Apolipoprotien A‐II (g/L)	0.38 ± 0.02	0.34 ± 0.02	0.101
Apolipoprotein B (g/L)	0.83 ± 0.06	0.76 ± 0.03	0.038
VLDL‐apoB (mg/L)	77.2 ± 0.95	56.6 ± 4.9	0.010
Apolipoprotein(a), nmol/L	86.4 ± 21.8	86.6 ± 21.6	0.884
Lipoprotein(a) (g/L)	0.26 (0.16–0.43)	0.24 (0.14–0.42)	0.205
Glucose (mmol/L)	5 (19 ± 0.10	−.32 ± 0.11	0.122
Insulin (mU/L)	7.74 ± 0.95	8.77 ± 0.89	0.217
HOMA score	1.79 ± 0.23	2.08 ± 0.21	0.137

*Note*: Values are mean ± SEM or geometric mean (95% confidence intervals); the values of clinical, biochemical and hemodynamic characteristics were determined at the end of each 8 week treatment period.

### 3.3 Treatment effect on cholesterol efflux capacity

**FIGURE 1:**
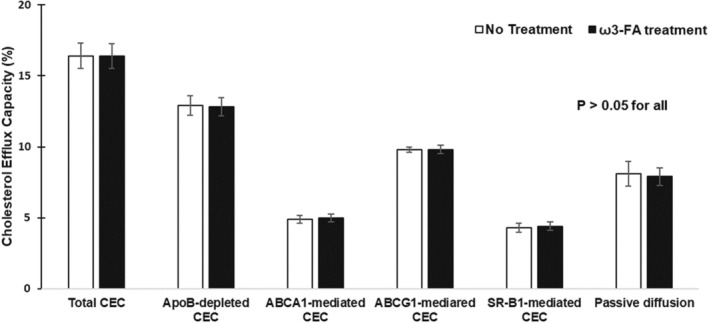
Effect of omega‐3 fatty acid supplementation on cholesterol efflux capacity in the FH patients.

Figure 1 shows the effect of ω‐3FAs on CEC in the subjects. Compared with no treatment period, ω‐3FAs did not significantly altered whole plasma (16.4% ± 0.9% vs. 16.4% ± 0.9%), apoB‐depleted (12.9% ± 0.7% vs. 12.8% ± 0.6%), ABCA1‐mediated (4.9% ± 0.3% vs. 5.0% ± 0.3%), ABCG1‐mediated (9.8% ± 0.2% vs. 9.8% ± 0.3%) and SR‐BI‐mediated CEC (4.3% ± 0.3% vs. 4.4% ± 0.3%) and passive diffusion‐mediated CEC (8.1% ± 0.7% vs. 7.9% ± 0.6%) in the FH subjects (*p* > 0.05 for all).

## 4 DISCUSSION

Our principal finding was that FH patients have impaired ex vivo capacity to promote cholesterol efflux from macrophage, but that this abnormality is not significantly corrected by ω‐3FA treatment.

### 4.1 Previous studies

Several experimental studies have consistently demonstrated that ω‐3FAs promote the RCT mechanism by stimulating faecal sterol excretion.^11^, ^12^ However, the effect of ω‐3FAs on cellular cholesterol efflux is unclear, with one study showing enhanced effect using human macrophages and other studies reporting no stimulation of cholesterol efflux from macrophage.^11^, ^12^, ^13^ EPA and DHA also appears to have diverging effects on cholesterol efflux from macrophage in mice and humans. The effects of ω‐3FA treatment on cellular cholesterol efflux have not yet formally been reported in humans. Only one study reported that 30 days of treatment with ω‐3FAs (1125 mg EPA/875 mg DHA daily) increased ABCA1‐mediated CEC in seven subjects, but the results were only communicated as an abstract.^14^ We have extended previous studies by investigating the effect of a higher dose of ω‐3FAs on cellular CEC in 20 FH patients.

### 4.2 Effect of ω‐3FAs on cholesterol efflux capacity

HDL particles are critical acceptors of cholesterol from lipid‐laden macrophages through an interaction with its phospholipid content, cellular receptors, lipid transfer proteins, lipases and apolipoprotein (apo) B‐100 containing lipoproteins.^3^, ^4^ HDL‐phospholipid content has also been shown as a major factor determining CEC. ABCA1 mediates cellular free cholesterol efflux to lipid‐poor apoA‐I whereas ABCG5 and SR‐BI preferentially promotes the efflux of cellular cholesterol to mature apoA‐I and/or apoA‐II containing HDL particles. There are other mechanisms or factors governing cholesterol efflux that may be affected by the latter stages in RCT. These include the availability of apoB‐100‐containing lipoproteins to accept cholesteryl esters from HDL particles which allows the plasma system to maintain the capacity of HDL to take up free cholesterol in HDL particles constant.^15^ We have previously reported that total CEC was positively associated with plasma level of apoB‐containing lipoproteins in men.^15^ We have also shown that the reduction in the capacity of plasma to effect cholesterol efflux was associated with the corresponding decrease in the concentration of apoB‐100 containing lipoproteins with statins.^9^ In the present study, we did not observe any significant effect of ω‐3FAs on cellular cholesterol efflux. Several reasons may account for it. First, ω‐3FAs did not alter on plasma HDL‐cholesterol, apoA‐I and apoA‐II levels. This suggests that ω‐3FAs may not beneficially affect cellular cholesterol efflux by influencing HDL remodelling in our patients. Second, ω‐3FAs reduced apoB particles in plasma. As discussed earlier, this effect may in turn offset any direct or indirect stimulation of CEC with ω‐3FAs. However, this speculation merits further investigation.

### 4.3 Study limitations

The sample size was small. Hence, the findings need to be interpreted with caution and confirmed in a larger population. We did not specifically compare the capacity of plasma to efflux cholesterol in FH patients with age and gender matched controls. Hence, we cannot exclude the possibility of these confounding effects. However, our findings were not altered by age and gender (data not shown). A formal case–control study matched for age and gender is required to confirm our observations. We did not study the differential effects of EPA and DHA on cellular cholesterol efflux. In view of the differential effects of EPA and DHA on lipid metabolism,^6^ it may be of interest to examine the role of pure DHA and EPA on cellular cholesterol efflux. Measurement of lipoprotein lipase, hepatic lipase, cholesteryl ester transfer protein and phospholipid transfer protein, as well as HDL‐phospholipid content may also help to formally corroborate our findings. It is known that the enrichment of membranes with ω‐3FAs can affect membrane protein structure and function.^6^ Our results might have been different had we evaluated the capacity to promote cholesterol efflux with macrophages isolated from the patients. Whether longer term treatment with ω‐3FAs increases CEC remains to be investigated.

## 5 CONCLUSION

If untreated, FH confers an extremely high risk of premature CAD.^1^ Although the dose of ω‐3FA employed in the present study can significantly and effectively lower in plasma triglyceride and blood pressure.^8^ Our data suggest that ω‐3FAs do not improve the capacity of plasma to efflux cholesterol from peripheral cells in the initial stage of RCT. Whether ω‐3FAs improve RCT in the latter stages by promoting hepatobiliary cholesterol excretion merits further investigation by detailed HDL kinetic and cholesterol balance studies. Future studies should also examine the additive effects of HDL‐raising agents, such as fibrates, to ω‐3FAs and their effects on cellular cholesterol efflux.

## AUTHOR CONTRIBUTIONS

Dick C. Chan analysed the data and wrote the manuscript. Gerald F. Watts and Dick C. Chan lead and designed the study, analysed data and edited manuscript. Qidi Ying and Jing Pang supervised data collection and edited the manuscript. Annalisa Ronca and Elda Favari collaborated with measuring cholesterol efflux capacity. Mikaël Croyal collaborated with measuring plasma apolipoprotein concentrations with LC–MS/MS. All authors contributed to the drafting and editing of the review, as well as subsequent revisions.

## FUNDING INFORMATION

This study was supported by a grant from NHMRC (1028883).

## CONFLICT OF INTEREST STATEMENT

Dick C. Chan, Annalisa Ronca, Qidi Ying, Mikaël Croyal and Elda Favari: None to disclose. Gerald F. Watts: reports funding or honoraria from Amgen, Pfizer, Esperion, Arrowhead, Regenron, Sanofi, AstraZeneca, Novartis.

## Supporting information


Data S1.
