# Supplementary material for: Does fish oil supplementation increase cholesterol efflux capacity in familial hypercholesterolaemia?
Source: Eur J Clin Invest. 2023 Jun 29;53(10):e14048. doi: 10.1111/eci.14048 (PMC10909456; doi:10.1111/eci.14048)

Supplementary Material

Subjects

Twenty-two patients with FH aged 18-70 years [body mass index <40 kg/m^2] were recruited from the Lipid Disorders Clinic at Royal Perth Hospital. Diagnosis of FH was defined by the Dutch Lipid Clinic Network criteria (DLCN) score >8 (definite FH) and/or the presence of a pathogenic variant causative of FH. Of the 22 eligible subjects, one withdrew consent before completing the first intervention period, and another was withdrawn because of an adverse event related to new onset atrial fibrillation that resolved spontaneously within 24 hours. Of the remaining 20 subjects, none had *APOE2/E2* genotype, proteinuria, creatininaemia (>120 µmol/L), hypothyroidism, intolerance to fish oil or abnormal liver enzymes (alanine aminotransferase >120 U/L for men and >90 U/L for women). None reported a cardiovascular event within six months prior to the study, or was taking fish oil supplementation or anti-diabetic medication. This study was approved by the Human Research Ethics Committee of the Royal Perth Hospital, and written informed consent was obtained from all subjects.

Clinical Protocol

This study was a randomized, crossover intervention trial. All eligible patients were on statins (or statin plus ezetimibe) at recruitment and throughout the duration of the study. All patients entered a 4-week run-in diet stabilizing period, at the end of which they were randomized to one of the two groups, no treatment or an 8-week treatment period of 4 g/d ω3FAs (Omacor® 46% EPA and 38% DHA in ethyl ester form, Abbott Products Pty Ltd) with an 8-week washout between each intervention period (Supplementary Figure 1). All subjects were reviewed fortnightly and requested to maintain their diet intake and usual level of physical activity. Dietary intake was

assessed for energy and major nutrients using at least two 3-day self-administrated dietary diaries. Diets were subsequently analysed using FoodWorks 10 (Xyris, Queenslandd Australia). Physical activity was assessed using a 7-day recall self-administrated questionnaire; energy expenditure (kJ/day) was calculated using the method described by Blair et al [1]. Dietary intake, alcohol and exercise were completed at the end of no treatment period and active treatment with ω3-FAs.

Measurement of ABCG1-mediated serum CEC

CHO-K1 cells stably expressing hABCG1 were generated as previously described [2]. Cells were plated in 24-well plates in HAM'S F-12 with 10% FCS. Parent and hABCG1-expressing cells were labeled for 24 h with [^{3}H]cholesterol, washed, and equilibrated for 90 min in serum-free medium prior to incubation in efflux medium containing BSA (1 mg/mL) in the presence of 1% of whole serum for efflux time (6 hours). To analyze the cellular [^{3}H]-cholesterol content in the cell monolayers, lipid extraction was carried out by adding 0.6 mL of 2-propanol. Cell media were then filtered through a 0.45 μm filter to remove floating cells, and radioactivity in the supernatant was determined using liquid scintillation counting (Opti-Fluor®, Perkin-Elmer). The efflux of cholesterol was measured as the ratio between the radioactivity released by cells exposed to sera in the culture medium and the cells at t_0. The ABCG1-mediated cholesterol efflux was then calculated as the difference between the percentage efflux from transfected cells minus the percentage efflux from CHO-K1 parent cells. ABCG1 expression was verified by the increase in efflux to 25 μg/ml HDL (used as extracellular acceptor) [3].

Measurement of SR-BI-mediated serum CEC

SR-BI-mediated cholesterol efflux was measured using Fu5AH rat hepatoma cells, a stable highly SR-BI-expressing cell line [4]. Cells were seeded in 48-well plates and grown in DMEM medium with 10% FCS for 1 day and then labeled with 2 µCi/mL [3H]cholesterol for 24 h in medium containing 1% FCS and an ACAT inhibitor (2 µg/mL). The ACAT inhibitor was added to ensure that all labelled cholesterol was present as free cholesterol. Cells were incubated with medium with or without BLT-1 (Block Lipid Transfer-1 10 µM, ChemBridge) for 2 h to evaluate the passive diffusion process or SR-BI-mediated and PD-mediated efflux, respectively. After this incubation, some wells were washed with PBS, dried, and extracted with 2-propanol; these cells provide baseline (t_0) values for total [^{3}H]cholesterol content. Cells were then washed with PBS and incubated for 4 hr in the presence of 2% (v/v) of the subjects' sera under examination. Cell media were then filtered through a 0.45 µm filter to remove floating cells, and radioactivity in the supernatant was determined by liquid scintillation counting. Cholesterol efflux was calculated as: (cpm in medium at 4h/cpm at time 0) x 100. The SR-BI activity was assessed by measuring the efflux to 25 µg/ml HDL (used as extra-cellular acceptor).

Biochemical measurements

Fasting blood samples were collected at the end of each treatment period. Briefly, fasting whole venous blood samples collected in EDTA were immediately centrifuged at 1500 x g for 15 min at 4°C. Plasma was collected and stored at -80°C. Plasma lipid and glucose concentrations were measured using enzymatic methods (Roche Diagnostics Australia, Castle Hill, NSW, Australia). LDL-cholesterol was estimated by the Friedewald calculation. Total plasma apoB-100 concentrations were determined

by immunonephelometry (Dade Behring BN2 nephelometer). TRL fraction was isolated from 3.5 mL plasma by ultracentrifugation (Optima XL-100K, Beckman Coulter, Australia) at d < 1.006 g/mL (40,000 rpm, 16 h, 4°C). Plasma VLDL-apoB-100 was measured in the TRL fraction using an ELISA kit (Mabtech, Nacka, Sweden). The kit is specific for apoB-100 and does not recognize apoB-48. Total lipoprotein(a) [Lp(a)] mass concentration was measured by an automated latex enhanced immunoassay (Quantia Lp(a) assay and standards, Abbott Laboratories, Abbott Park, IL). Quantification of plasma A-I, A-II and apo(a) were determined by liquid chromatography-tandem mass spectrometry (LC-MS/MS) as described previously [5]. Fasting insulin was measured using chemiluminescent immunometric assay (Abbott Diagnostics, North Ryde, NSW, Australia), and insulin resistance was estimated using homeostasis model assessment (HOMA score). Plasma EPA and DHA levels were measured by gas chromatography mass spectrometry [6].

References

1. Blaire SN. How to assess exercise habits and physical fitness. In: Matarazzo, ed. Behavioural Health. New York: John Wiley and Sons. 1984: 424-427.
2. Jessup W, Gelissen IC, Gaus K, Kritharides L. Roles of ATP binding cassette transporters A1 and G1, scavenger receptor BI and membrane lipid domains in cholesterol export from macrophages. Curr Opin Lipidol 2006;17:247-257.
3. Favari E, Calabresi L, Adorni MP, Jessup W, Simonelli S, Franceschini G, Bernini F. Small discoidal pre-beta1 HDL particles are efficient acceptors of cell cholesterol via ABCA1 and ABCG1. Biochemistry. 2009;48:11067-11074.

4. Rothblat GH, de la Llera-Moya M, Favari E, Yancey PG, Kellner-Weibel G. Cellular cholesterol flux studies: methodological considerations. Atherosclerosis. 2002;163:1-8.
5. Blanchard V, Garçon D, Jaunet C, Chemello K, Billon-Crossouard S, Aguesse A, Garfa A, Famchon G, Torres A, Le May C, Pichelin M, Bigot-Corbel E, Lambert G, Cariou B, Hadjadj S, Krempf M, Bach-Ngohou K, Croyal M. A high-throughput mass spectrometry-based assay for large-scale profiling of circulating human apolipoproteins. J Lipid Res. 2020; 61:1128-1139.
6. Mori TA, Burke V, Puddey IB, Watts GF, O'Neal DN, Best JD and Beilin LJ. Purified eicosapentaenoic and docosahexaenoic acids have differential effects on serum lipids and lipoproteins, LDL particle size, glucose, and insulin in mildly hyperlipidemic men. Am J Clin Nutr. 2000;71:1085-1094.

Figure 1 Study design

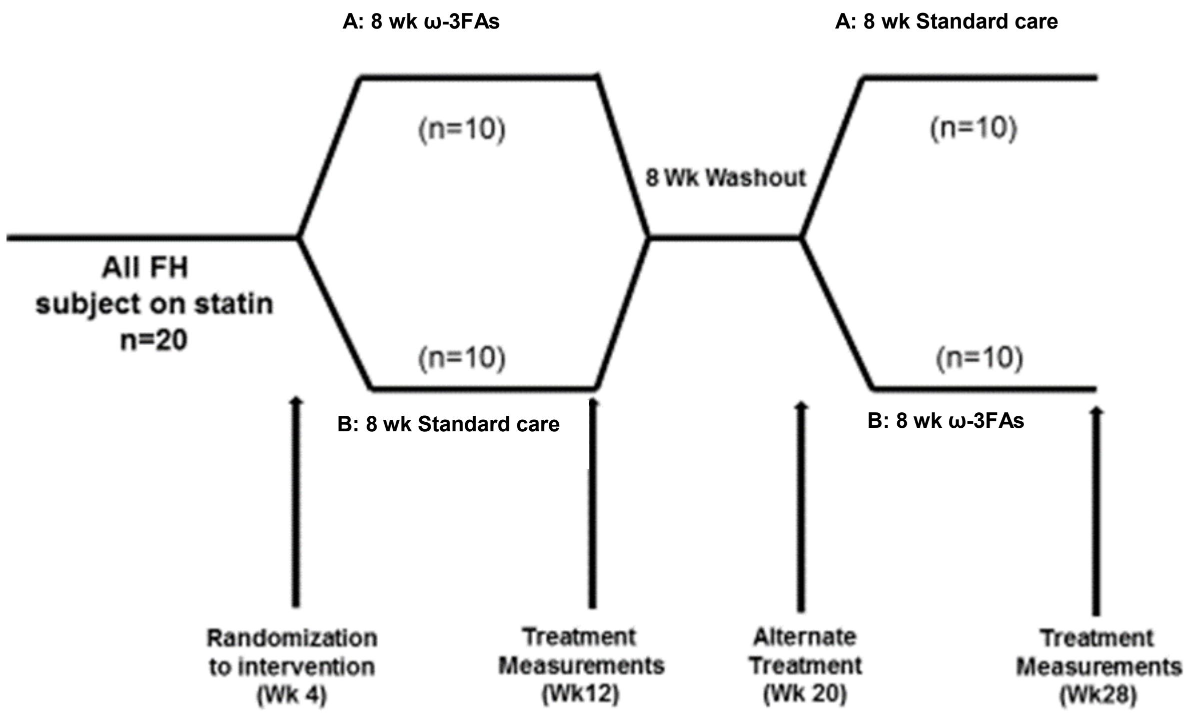

Supplement: Supplementary file 1 — Data S1. [file ECI-53-e14048-s001.pdf]
